# Delirium in older hospitalized patients—A prospective analysis of the detailed course of delirium in geriatric inpatients

**DOI:** 10.1371/journal.pone.0279763

**Published:** 2023-03-16

**Authors:** Skadi Wilke, Edgar Steiger, Tanja L. Bärwolff, Justus F. Kleine, Ursula Müller-Werdan, Adrian Rosada

**Affiliations:** 1 Department of Geriatrics, Charité-Universitätsmedizin Berlin, Berlin, Germany; 2 Central Research Institute of Ambulatory Health Care in Germany (Zi), Berlin, Germany; 3 Department of Neuroradiology, Charité-Universitätsmedizin Berlin, Berlin, Germany; Fukuoka University, JAPAN

## Abstract

**Background:**

Delirium in older hospitalized patients (> 65) is a common clinical syndrome, which is frequently unrecognized.

**Aims:**

We aimed to describe the detailed clinical course of delirium and related cognitive functioning in geriatric patients in a mainly non-postoperative setting in association with demographic and clinical parameters and additionally to identify risk factors for delirium in this common setting.

**Methods:**

Inpatients of a geriatric ward were screened for delirium and in the case of presence of delirium included into the study. Patients received three assessments including Mini-Mental-Status-Examination (MMSE) and the Delirium Rating Scale Revised 98 (DRS-R-98). We conducted correlation and linear mixed-effects model analyses to detect associations.

**Results:**

Overall 31 patients (82 years (mean)) met the criteria for delirium and were included in the prospective observational study. Within one week of treatment, mean delirium symptom severity fell below the predefined cut-off. While overall cognitive functioning improved over time, short- and long-term memory deficits remained. Neuroradiological conspicuities were associated with cognitive deficits, but not with delirium severity.

**Discussion:**

The temporal stability of some delirium symptoms (short-/long-term memory, language) on the one hand and on the other hand decrease in others (hallucinations, orientation) shown in our study visualizes the heterogeneity of symptoms attributed to delirium and their different courses, which complicates the differentiation between delirium and a preexisting cognitive decline. The recovery from delirium seems to be independent of preclinical cognitive status.

**Conclusion:**

Treatment of the acute medical condition is associated with a fast decrease in delirium severity. Given the high incidence and prevalence of delirium in hospitalized older patients and its detrimental impact on cognition, abilities and personal independence further research needs to be done.

## Introduction

Delirium in older hospitalized patients (> 65 years) is a common clinical syndrome defined by the Diagnostic and Statistical Manual of Mental Disorders, 5^th^ edition (DSM-5) [1] as an acute and fluctuating disturbance in attention and awareness as a direct consequence of a physiological condition (i.e. medical conditions, substance intoxication, or withdrawal, exposure to a toxin [1]. Although well discussed in the literature, delirium remains unrecognized in many patients. Estimations of incidence and prevalence of delirium vary a lot. On admission 18–35% (prevalence) of older hospitalized patients show symptoms of delirium and 11–29% (incidence) develop a delirium in the course of their hospital stay [2], whereas certain patient groups are more susceptible than others–especially patients with cancer, any terminal illness and patients after surgery are more vulnerable [3] As hospitalized geriatric patients frequently suffer from multimorbidity, delirium is multifactorial [4] Decades of research could already show a complex interaction between predisposing and precipitating factors [2]. Combined with precipitating factors predisposing factors are presumably triggering inflammatory processes and yielding to imbalances in neurotransmitter levels [5] As an early marker for inflammation or infection, laboratory parameters such as the C-reactive protein (CRP) and white blood cell count (WBC) levels are used [6]. Among others, elevated CRP [7] and WBC levels have been found in patients with delirium symptoms [8]. Structural brain risk factors include atrophy and white matter hyperintensities (WMH) [9].

There is evidence for delirium being a strong predictor for cognitive decline and the incidence of dementia in later life as both an independent [10] and accelerating [11] risk factor. In addition, some longitudinal studies reported an association between delirium in connection with intensive care treatment, hip surgery and hematopoietic cell transplantation and lower abilities in basic activities of daily living (ADL), worsening in quality of life at 6-month-follow-up [12], loss of personal independence and even showing a link to symptoms of depression and post-traumatic stress [12–15].

Whereas most studies concentrated on postoperative delirium and its risk factors [16, 17], only a few described the clinical course and outcome of delirium in in-hospital older patients, showing adverse effects in cognitive and functional status [18, 19].

When it comes to delirium in the non-postoperative setting, symptoms are frequently unrecognized [20] or misdiagnosed as other psychiatric disorders [21]. However, in accordance with the DSM-5, the diagnosis of delirium has to remain uncertain until sufficient information about the patient’s baseline mental status and acute changes from a competent proxy has been acquired [1]. The first clinical impression can only be an indication. A better understanding of delirium, its course and association with clinical characteristics and cognitive functioning could be helpful for diagnosis and treatment.

Here, we aimed to conduct an exploratory study to describe the course of delirium severity and symptoms and related cognitive functioning in geriatric inpatients in association with demographic and clinical characteristics (e.g. prediagnosed dementia), generally hypothesizing that 1) patients with severe delirium symptoms show poorer cognitive performance in non-delirium specific tests and 2) the overall symptom severity regresses and the cognitive functioning improves over the course of rehabilitative treatment also in non-delirium specific tests. Furthermore, we aimed to determine 3) possible risk factors such as brain imaging markers, hypothesizing that conspicuous findings are associated with poorer cognitive performance and more severe delirium symptoms.

## Methods

### Data collection and sample

A prospective observational chart review study was conducted in order to examine the course of delirium in geriatric patients. All data were extracted from digital medical records. Inclusion criteria for data extraction: 1) age over 60 years, 2) hospital admission between March 2017 and July 2018, 3) initial delirium diagnosis by a trained psychologist and 4) hospital stay of at least 7 days.

Patient data were pseudonymized.

### Study design and measures

Over the course of an inpatient stay (~ two weeks) in a 30-bed geriatric unit of an university-teaching hospital, all admitted patients received a psychological baseline examination including cognitive testing using the Mini-Mental-Status-Examination (MMSE) [22] and emotional assessment using the Geriatric Depression Scale (GDS) questionnaire [23] as standard procedure. By default, conspicuous findings according to the DSM-5 regarding delirium confirmed by a proxy interview (regarding pre-clinical cognitive and psychiatric status, drug and alcohol abuse) were leading to further assessments using the Delirium Rating Scale Revised 98 (DRS-R-98) [24] and further follow-up examinations (MMSE + DRS-R-98) planned in a 2-3-day rhythm. T0 was admission to the geriatric ward; T1 was the time delirium was the first time assessed (not equal to delirium-onset as information on pre-hospital status was incomplete to not existing); T2 was set as time of the second assessment 2–3 days after T1; T3 was set as time of the third assessment 2–3 days after T2. To reduce dependence of daytime all assessment were performed in the morning period after breakfast. Each visit consisted of an assessment of cognitive functioning (MMSE) and delirium symptoms (DRS-R-98) in order to examine the course of delirium.

All patients also received activating nursing care, daily occupational therapeutic and physiotherapeutic sessions by trained personnel.

At the same time, medical conditions like bacterial infections, uncontrolled metabolic conditions or adverse drug reactions were diagnosed and adequate medical treatment was initiated. If indicated patients additionally received medication for delirium treatment: Melperone if sleep disturbance was very prominent, Risperidone if agitation was very prominent according to clinical assessment by the treating physician.

As further baseline and outcome measures, the patients’ individual functional status was assessed at admission and at discharge by a trained nurse using the Barthel Index (BI) [25].

Moreover, demographic data such as age, sex and place of residence were extracted.

### Mini-Mental-Status-Examination (MMSE)

The MMSE is a reliable and widely used instrument in research and the clinical context in order to measure cognitive abilities including orientation, verbal memory, attention, language and visuospatial praxis [22] A global cut-off score of 24 or below (out of 30 points) indicates cognitive impairment.

### Delirium Rating Scale Revised 98 (DRS-R-98)

Presence and severity of delirium were assessed using the DRS-R-98, a 16-item scale with 13 severity items and three diagnostic items administered by a trained psychologist. It was chosen because it covers a broad range of symptoms, which may occur in delirium including symptoms of hypoactivity. The DRS-R-98 scale is a reliable tool to assess changes in individual symptom severity over time where each item is rated on a scale from 0 to up to 3 points. Higher scores indicate more severe delirium symptoms. Cut-off scores were used to determine the presence of delirium (≥17.75) and symptom severity (≥15.25) [24]. Trzepacz and colleagues (2001) report high sensitivity and specificity in distinguishing delirium from dementia [24] The DRS-R-98 includes the following items: 01: sleep-wake cycle disturbance, 02: perceptions and hallucinations, 03: delusions, 04: lability of affect, 05: language, 06: thought process abnormalities, 07: motor agitation, 08: motor retardation, 09: orientation, 10: attention, 11: short-term memory, 12: long-term memory, 13: visuospatial ability, 14: temporal onset of symptoms, 15: fluctuation of symptom severity, 16: physical disorder.

Furthermore, a competent proxy was interviewed using a semi-structured approach in order to obtain more information about the respective patient’s preclinical mental status and predisposing factors.

### Delirium etiology

In order to evaluate the possible etiology of delirium, a physician reviewing each patient’s chart extracted four categories: a) post-operative, b) drug, c) infection, d) other.

These categories were chosen after reviewing and grouping the specific etiologies of the examined patients and do not represent the full broad heterogeneity of the origin of the illness of delirium.

### Laboratory parameters—CRP and WBC

For most patients, laboratory parameters CRP and WBC as surrogates for inflammation, albumin as surrogate for nutritional status, creatinine as surrogate for kidney function and sodium and potassium for electrolyte status were analyzed by the hospital laboratory. These laboratory parameters could be extracted for three time points (±1 day) from the digital medical records.

### Barthel Index (BI)

The BI was conducted at admission and at discharge in order to assess the patients’ abilities in basic ADL ranging from 0 defined as complete dependence to 100 defined as complete independence.

### Brain imaging biomarkers

All patients but two received routine structural computed tomography (CT) (N = 18) or magnetic resonance imaging (MRI) (N = 9) scans of the brain during their hospital stay. Blinded to the medical condition, a trained neuroradiologist reviewed these scans retrospectively on the following visual rating scales: Global cortical atrophy (GCA) [26], Scheltens scale [27], Wahlund scale [28], Fazekas scale [29], Koedam scale [30] (see S1 File for further details).

## Statistical methods

Statistical analysis was conducted with the R Software [31]. We explored the data with correlation analyses and different linear mixed-effects (LME) models to investigate the temporal characteristics of delirium symptoms and other characteristics of delirium.

At the beginning, we derived general characteristics of the data and in regards of missing values, we used Little’s missing-completely-at-random (MCAR) test to assess if the data satisfies the MCAR hypothesis [32].

For correlation analysis, we analyzed the general associations between different test scores (DRS-R-98, MMSE, and BI) on different assessment days, along with demographic characteristics age, gender, and prediagnosed dementia, as well as treatment medication (Melperone, Risperidone or any). Afterwards, we inspected correlation matrices for the three assessment days to explore the associations between individual DRS-R-98 symptom scores, their sum (total score), MMSE score, laboratory parameters CRP and WBC, and demographic characteristics as well as medication. Furthermore, correlations between structural brain imaging biomarkers and the MMSE and DRS-R-98 score on day one were conducted respectively. All correlations were calculated as Spearman correlations.

After these preliminary association explorations, we calculated effects on observed scores (DRS-R-98 and MMSE) in a multivariate setting, where we controlled for confounding variables with LME models (R package lme4) [33]. We standardized predictor variables by applying Gelman’s recommendations [34]. For all three LME modelling strategies respectively, following closely the guidelines of Harrison and colleagues [35], we examined model assumptions and identified the most appropriate models (see S1 File). We give significance results (p-values, confidence intervals) for the respective final models [36]. Choosing the most parsimonious model with the Akaike information criterion (AIC) served here as a sensitivity analysis of model selection [37].

In the first model, we investigated the general temporal dynamics of the total DRS-R-98 score and the influence of demographic factors, medication, and laboratory parameters. In the second model, we analyzed the temporal dynamics of individual DRS-R-98 symptoms. Finally, in the third model, we explored the relationship between MMSE and delirium symptoms as measured by DRS-R-98 rating scale in order to investigate their impact on cognition. For more detailed descriptions of fixed and random effects in the LME models, see the S1 File.

The study was approved by the ethics committee of the Charité—Universitätsmedizin Berlin (EA4/179/20). All patients provided informed written consent to have data from their medical records used in research.

Due to the non-interventional nature (all procedures were part of the standard patient survey) of this observational study, the need for further informed consent was waived by the ethics committee. All patients or their close relatives, if the patient was not capable of being informed by the end of the hospital stay, were informed about the results and the implications of their clinical surveys. All analyses were conducted with deidentified data.

## Results

42 patients met the DSM-5 criteria for delirium [1]. Data from eleven patients were excluded from further analyses due to shortened hospital stays and early relocation. Thus, overall 31 patients (82 years [66, 92] (mean [range])) met the inclusion criteria and were included in further analyses. See Table 1 for sample characteristics.

**Table 1:** Demographic and clinical characteristics of the sample.

*Characteristics*		
Sex	15, male, 15 female	
Age (mean, [range])	82.3 years [66,92]	
Duration hospital stay (mean, [range])	22.7 days [7,61]	
BI adm. (mean (SD))	32.17 (16.95)	
BI disch. (mean (SD))	42.69 (24.26)	
*Main Diagnoses on Admission (n (%))*		
Fall and fracture	8 (26.7%)	
Infection	5 (16.7%)	
Cardiac	3 (10%)	
Delirium	3 (19%)	
Other*	10 (33.3%)	
*Predisposing Factors on Admission (n (%))*		
Dementia	11 (36.7%)	
MCI	6 (20.0%)	
Depression	13 (43.3%)	
Diabetes mellitus	15 (50.0%)	
Hypothyreosis	6 (20.0%)	
Delirium triggering medication**	14 (46.7%)	
*Delirium Etiology (n (%))*		
Infection	17 (56.7%)	
Post-operative	4 (13.3%)	
Drugs	3 (10.0%)	
Other***	6 (20.0%)	
*Received Medication for Delirium treatment (n(%))*	15 (50%)	
*Melperone*	12 (40%)	
*Risperidone*	6 (20%)	
*both*	3 (10%)	
*Laboratory Parameters* (mean (SD))	CRP in mg/L	WBC in 10^9^/L
t1	50.58 (54.13)	9.40 (4.18)
t2	45.02 (44.84)	9.35 (4.23)
t3	40.16 (47.14)	8.19 (4.03)
*Delirium and Cognition* (mean (SD))	DRS-R-98 total score	MMSE
t1	23.13 (4.95)	15.30 (6.33)
t2	18.05 (6.55)	18.64 (4.95)
t3	15.53 (6.81)	20.63 (3.66)
*Brain Imaging Biomarkers* (median [range])		
Fazekas-Score	2 [0–3]	
Sheltens-Score	2 [0–3]	
GCA	2 [1–2]	
Koedam-Score	1 [0–2]	
Wahlund-Score	8 [1–22]	

***Note***. BI adm. = Barthel Index at admission, BI disch. = Barthel Index at discharge, CRP = c-reactive protein, WBC = white blood cell count, DRS-R-98 = Delirium Rating Scale Revised 98, MCI = Mild Cognitive Impairment (DSM-5); MMSE = Mini-Mental Status Examination, t1 = assessment 1, t2 = assessment 2, t3 = assessment 3.

* Other main diagnoses (cancer, polyneuropathy, drug related, acute renal failure, stroke, gastrointestinal bleeding)

**Delirium triggering medication (opiates, benzodiazepines, cortisone)

*** Other Delirium etiology (uncontrolled Metabolic conditions Hyperglycemia; Hypothyreosis).

Out of 31 individuals total, we removed one patient’s data from all analyses since assessment two and three happened three weeks after the first assessment. 30 individuals were followed through the initial and first follow-up assessment, finally 28 individuals were followed through all study visits (three time points). Thus, our data for analysis consists of all assessments that happened within one week of the first (baseline) assessment.

We observed missing values for MMSE scores on 8 time points for 7 different patients, 5 missing values of the BI for 5 different patients at discharge, as well as some missing values for laboratory parameters CRP and WBC (3 missing values each, across 3 different patients and 4 days). Little’s MCAR test was not significant (p = 0.29) on the null hypothesis that data is missing completely at random. Thus, observations with missing values were left out of the respective LME models (complete-case analysis).

Mean age of patients was 82.3 years (standard deviation 6.6, range 66 to 92) and 50 percent of patients were female. Furthermore, 36.7 percent of patients had a prediagnosed dementia. Delirium occurred regularly in the first two weeks since hospital admission, but also developed several weeks after admission with decreasing probability of occurrence with progressing time. The median time of initial delirium diagnosis was 11 days (range 0 to 11) after hospital admission indicating that most patients were already showing delirium symptoms on admission to our geriatric ward, whereby most were caused by a bacterial infection (58.07%) (Table 1). A majority of the patients with delirium showed increased CRP levels (mean 50.58 mg/L, SD: 54.13 mg/L) and also elevated but still in range WBC levels (mean: 9.4 10^9^/L, SD: 4.18 10^9^/L). At the same time, levels of albumin, creatinine, potassium and sodium were in range. Some patients received Melperone or Risperidone as treatment medication: 12 patients received Melperone, 6 received Risperidone, and since 3 received both, for a total of 15 patients or 50% that received medication as part of their treatment. In 3 patients specific drugs (high doses of levodopa, prednisolone) were identified as main specific trigger for delirium. In these cases those drugs were lowered in dosage or discontinued if possible according to clinical assessment by the treating physician.

Furthermore, most patients with delirium preclinically lived on their own (58%) or in a shared household (35%) and a large group was postclinically discharged into the care of a nursing home (45%) after treatment. The mean BI at admission was 31.61, indicating severe dependency which improved at discharge with 41.48, but still indicating severe dependency in ADL.

**Fig 1:**
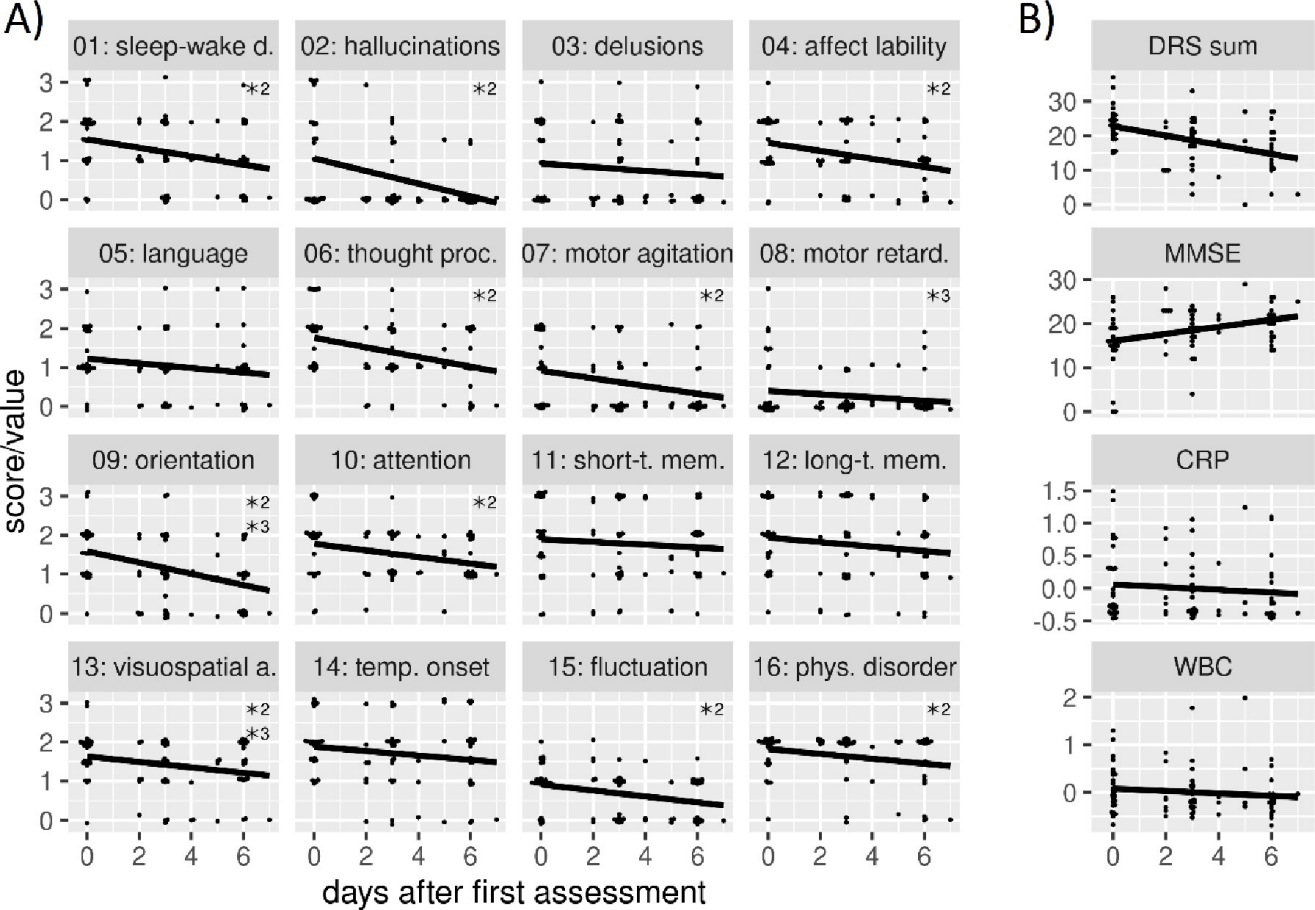
**A:** DRS-R-98 symptom scores at assessment times. Higher values indicate higher symptom severity. **B:** DRS-R-98 total score, MMSE, CRP and WBC at assessment times. *^2^ = significant effects in the second LME model, *^3^ = significant effects in the third LME model. DRS-R-98 = Delirium Rating Scale Revised 98, MMSE = Mini-Mental Status Examination, MMSE = Mini-Mental-Status Examination, CRP = C-reactive protein, WBC = white blood cell count.

Mean DRS-R-98 total scores varied considerably between time points (assessment 1: 23.7, assessment 2: 18, assessment 3: 15.5, overall: 19.1 with standard deviation 7 and range 0 to 37), but individual item scores varied differently between subjects and time points (Fig 1B). Patients with prediagnosed dementia show higher mean DRS-R-98 total scores at all three time points (assessment 1: 25.9 (5.7), assessment 2: 18.0 (5.4), assessment 3: 17.8 (5.7)) compared to patients with no prediagnosed dementia (assessment 1: 22.5 (4.1), assessment 2: 18.1 (7.3), assessment 3: 14.2 (7.2)). The DRS-R-98 total score as well as individual item scores consistently decreased by consecutive study visits, while MMSE increased (assessment 1: 15.3, assessment 2: 18.6, assessment 3: 20.6, overall: 18.2 with standard deviation 5.5 and range 0 to 29; Fig 1B). Patients with prediagnosed dementia show lower mean MMSE scores at all three time points (assessment 1: 11.2 (7.4), assessment 2: 16.6 (5.5), assessment 3: 19.3 (2.9)) compared to patients with no prediagnosed dementia (assessment 1: 17.7 (4.2), assessment 2: 19.8 (5.5), assessment 3: 21.3 (3.9)). Delirium severity in some symptoms (e.g. 02: hallucinations, 07: motor agitation and 08: retardation) seem to decrease faster, whereas some symptoms (e.g. short- and long-term memory) show a temporal stability or slower decrease (Fig 1A).

**Fig 2:**
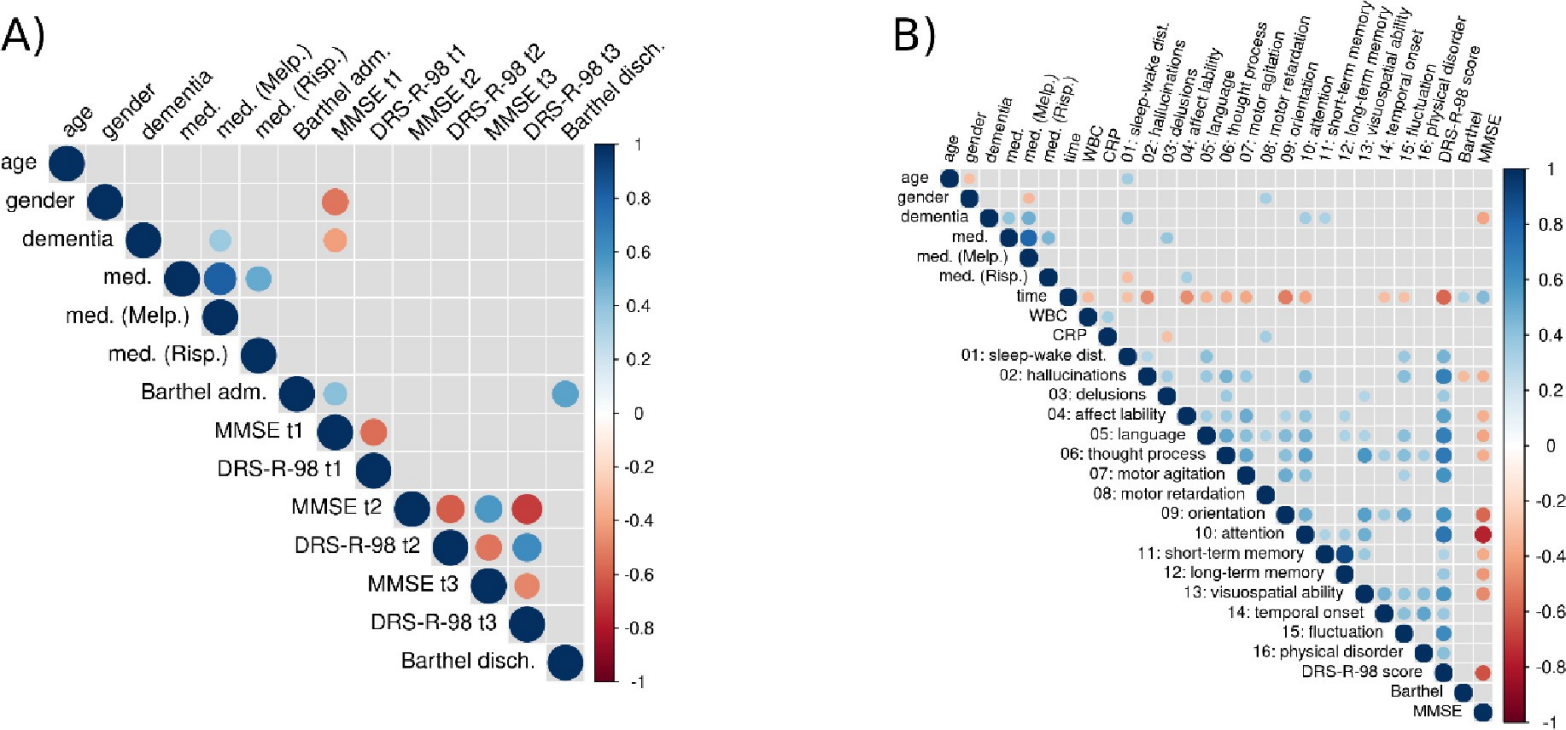
**A:** Correlation structure of test scores and demographic factors. Only correlations (Spearman) significant at 0.05 are shown. Dot size and color represent magnitude of correlation; darker color depicts stronger correlation. **B:** Correlation structure of DRS-R-98 symptoms and total score, MMSE score, and laboratory parameters: C-reactive protein and white blood cell count. Barthel adm. = Barthel Index admission, Barthel disch. = Barthel discharge, DRS-R-98 = Delirium Rating Scale Revised 98, MMSE = Mini-Mental Status Examination, t1 = assessment 1, t2 = assessment 2, t3 = assessment 3, CRP = C-reactive protein, WBC = white blood cell count.

Examining the correlations between scores of different tests on the three assessment days (Fig 2A), we observe strong negative correlations between MMSE and DRS-R-98 scores on the same day. DRS-R-98 scores of day 2 and 3 correlate with each other, as do MMSE test scores. MMSE and DRS-R-98 total scores from day 2 and day 3 correlate strongly with each other. BI scores on the first and final day show a strong correlation with each other, but only the initial BI score correlates to some degree with the first MMSE score. Male patients performed worse on MMSE assessments on day 1. Patients with prediagnosed dementia show lower MMSE scores on day 1, too, and were more often treated with Melperone.

Furthermore, significant negative correlations between the MMSE score (day 1) and the global Wahlund (r = -0.436, *p* = 0.033) and the Fazekas score (r = -0.496, *p* = 0.014) respectively were found, but not for the remaining visual rating scales (Table 2). No significant correlations between brain imaging markers and delirium severity were found (Table 2).

**Table 2:** Spearman rank order correlation for MMSE/DRS-R-98 total score (day 1) and the brain imaging biomarkers.

Variable	GCA	global Wahlund	global Fazekas	Scheltens	Scheltens	Koedam	Koedam
				left	right	left	right
MMSE	0.03	**-0.44** *	**-0.50** *	-0.07	-0.02	-0.09	0.09
DRS-R-98	-0.1	0.06	0.21	-0.17	-0.37	-0.18	-0.16

*Note*. GCA = global cortical atrophy, MMSE = Mini-Mental State Examination, DRS-R-98 = Delirium Rating Scale Revised 98.

*p ≤ 0.05

Next, we compared pairwise correlations of demographic variables, medication, laboratory parameters, individual DRS-R-98 symptom scores, and overall DRS-R-98/MMSE scores across all days. Fig 2B shows aggregated results over all three assessment days (see S1–S3 Figs for separate assessment days).

Across all three assessment days, the same DRS-R-98 symptoms correlate significantly with the DRS-R-98 total score: hallucinations, affect lability, language, thought process, motor agitation, attention, visuospatial ability, and fluctuation. Symptom scores of short-term and long-term memory are expressed highly similar. The DRS-R-98 symptom score for attention correlates negatively with MMSE score on all three days. The DRS-R-98 symptom score for delusion correlates negatively with laboratory parameters CRP and negatively with medication. Overall, we notice very similar patterns of correlation on all three days, and pairwise tests on differences between these correlation matrices were not significant, this indicates that overall correlation patterns (as shown in Fig 2B) did not change in the observation period.

Our first LME model framework was on the DRS-98 total score as outcome. Diagnostic plots showed no systematic departure from model assumptions (see S4 Fig), but the QQ plot of random effect quantiles suggests that there might be a grouping of patients that we could not describe with our available variables. The most parsimonious model contained only time (measured in days after first assessment) as a fixed effect. Time showed a significant negative effect (Table 3), that is, with each subsequent day we observed a decrease of the DRS-R-98 total score by about -1.42, while individual characteristics of patients (age, gender, dementia) as well as laboratory parameters, medication, and temporal interaction effects did not explain significant differences in DRS-R-98 total scores.

**Table 3:** Results of final LME model on DRS-R-98 total score.

Variable	estimate	CI-95% lower	CI-95% upper	p-value	sign.
(Intercept)	23.03	20.86	25.23	0	
time_day	-1.42	-1.90	-0.92	0	

Note. LME = Linear mixed-ffects model, DRS-R-98 = Delirium Rating Scale Revised 98, CI = confidence interval, sign. = significance.

Analysis of conditional and marginal R-squared values (see S3 Table) shows that time and individual effects explain less than 50 percent of variation in the observations within the chosen model in total.

Next, we checked if there are specific effects for individual DRS-R-98 symptom scores. To this end, we used DRS-R-98 symptom scores as outcome in an LME model. Diagnostic plots (see S5 Fig) showed no systematic departure from model assumptions. The final chosen model (see S1 Table) includes random slopes for CRP and WBC values, as well as fixed effects individual symptoms and their temporal interactions, while demographic variables, medication, and fixed effects for laboratory parameters fail to contribute significantly.

Overall, DRS-R-98 symptoms of hallucination, delusion, agitation, motor retardation, and symptom fluctuation have significantly smaller scores compared to other symptoms. Likewise, symptoms short-/long-term memory and symptom onset have significantly larger overall scores compared to other symptoms. Temporal decrease in symptom scores was significant for all symptoms but symptoms delusion, language, motor retardation, short-/long-term memory, and symptom onset.

Our final analysis connected DRS-R-98 and MMSE assessments. Diagnostic plots (see S6 Fig) showed no systematic departure from model assumptions but four assessments of MMSE had very low scores, which lead to visible deviations. The most parsimonious LME model with MMSE score as outcome was the model that included DRS-R-98 symptoms, demographic variables, and their temporal interactions (see S2 Table).

DRS-R-98 symptoms motor retardation, orientation, and visuospatial ability have significant negative effects on final MMSE scores. Furthermore, with this analysis, we identified two groups of patients that showed significant increases of MMSE scores by time. Male patients had approximately -4.45 points less at first assessment, but increased their MMSE score by approximately 0.93 points with every day after the first assessment. Similarly, patients with prediagnosed dementia had an estimated -4.65 points less at first assessment, but increased this score by 0.81 with every day subsequently. In addition, the chosen model is able to explain almost 70 percent of variation within the MMSE scores with the fixed effects only, and almost 80 percent by including the individual patient’s effects (see S3 Table).

## Discussion

Severity of delirium symptoms is decreasing over the course of treatment independent of symptom, age, sex or preclinical cognitive status. Within one week of treatment, the mean symptom severity fell under the predefined cut-off. Higher values in the DRS-R-98 scale are associated with poorer performance in the MMSE. At the same time, cognitive functioning is improving over time, whereas prediagnosed dementia and the male sex are associated with poorer performance in the first MMSE and show a large recovery rate. Severe symptom expressions in some symptoms (hallucinations, affect lability, impaired language, thought process, attention, visuospatial ability, motor agitation and symptom fluctuation) are associated with overall delirium severity. Temporal decrease in symptom scores was significant for all symptoms but delusion, language, motor retardation, short- and long-term memory. Furthermore, sleep-wake cycle disturbances and motor retardation have significant negative effects on final MMSE scores. In addition, impaired attention is associated with overall poorer performance in the MMSE. Negative associations between the MMSE and Wahlund and Fazekas score indicate neuroradiological correlates for worse cognitive functioning, while no association was found between those and the DRS-R-98 total score. Also, no association between CRP, WBC and delirium severity was found. Even though 50% of patients received medication to treat delirium we could not explain significant differences in DRS-R-98 total scores in connection with such medication via multiple regression analysis using a linear mixed model. Most likely our study is not suitable to assess the direct effects of each of the various interventions to treat delirium (like treating infection, discontinuing delirium triggering medication, usage of medication to treat delirium. After the hospital stay, proportionately fewer patients lived independently in their own or shared household and more patients lived in a nursing home comparing the pre- and post-clinical place of residence. This is a very relevant factor in social and economic point of view, as delirium seems to be a relevant component to the enforced relocation due to cognitive impairment. If this impairment is only temporary and some symptoms decrease slower- for example a temporary relocation to a nursing home could be a practical procedure which has to be elevated in future studies focusing on this topic.

Overall, the patients’ abilities in ADLs (BI) improved towards the end of the rehabilitative stay. Although the mean BI at discharge still indicates severe dependency.

The DRS-R-98 scale as used in the present study and others [38, 39] allows a detailed assessment of a broad range of delirium symptoms [24]. Furthermore, it has been shown that the DRS-R-98 scale is a reliable tool for discriminating between delirium and dementia [40].

In general, most studies regarding the course of delirium are conducted in surgical or anaesthetic settings in order to control for confounding variables. Only a few studies so far described the clinical course of delirium in older inpatients [18, 19, 39]. McCusker and colleagues (2003) assessed the clinical course of delirium also in a 2- to 3-day rhythm, but conducted further follow-ups after one, two, six and 12 months. They reported an overall decrease in the number of symptoms with impaired attention, orientation and memory being the most persistent symptoms at follow-ups regardless of the preclinical cognitive status (dementia vs. no dementia). This is in line with the findings of the present study, where an overall decrease in delirium symptom severity was found, but temporally stable short- and long-term memory deficits. This could be an indication for either preclinical cognitive impairment, as we did not have data regarding preclinical cognitive status other than the information the proxies have given us. Given a high amount of depressive patients, also this has to be acknowledged as a contributing factor for cognitive impairment. It could also indicate delayed cognitive improvements [41] or long-term cognitive decline as a result of delirium [10, 11].

Similar to our study, McCusker and colleagues found overall improved MMSE and BI scores, although only reported for the monthly follow-ups. Besides an overall increase in MMSE scores, we also found that patients with preclinical dementia performed poorer on day one as supported by findings of Trzepacz and colleagues (1998) but recovered faster, though the mean of the final MMSE score still indicates cognitive impairment (MMSE <24 points). Very interestingly, the recovery from delirium seems to be independent of preclinical cognitive status, which supports previous findings that more severe cognitive impairment (i.e. cognitive symptoms apart from other delirium symptoms) during an episode of delirium may be an indicator of dementia [40, 42].^⁠^ The temporal stability of some delirium symptoms (short-/long-term memory, language and motor retardation) on the one hand and on the other hand decrease in others (e.g. hallucinations, orientation, etc.) could also be an indication for pathology-related symptom profiles distinctive for dementia vs. delirium. Trzepacz and colleagues (2001, 1998) reported similar presentations of delirium symptoms in patients with and without dementia, however, pointing out that cognitive impairment is more pronounced in patients with delirium superimposed on dementia [24, 40]

Attention deficits are associated with poorer performance in MMSE. Grover and colleagues (2015) also found this association, hypothesizing detrimental effects of attention deficits on overall cognitive functioning [43]. Furthermore, our data shows that disturbances in sleep-wake cycles as a core symptom of delirium are associated with deficits in cognition. This is in line with several studies examining the influence of sleep on cognitive functioning in older adults [44] As a potentially modifiable risk factor, sleep disruption management and in association pharmacological and non-pharmacological interventions have been studied showing mixed results [45] Our data also suggests sex differences in the MMSE during delirium—male patients with delirium performed worse on the first assessment, which contradicts prior studies regarding sex differences in MMSE [46]. But as we did not control for differences in education and prior studies indicated an impact [47] this result has to be interpreted carefully.

Furthermore, poorer cognitive performance in the MMSE was associated with conspicuous brain imaging markers (Wahlund and Fazekas score) generally assumed to reflect cerebral white matter small vessel disease (SVD). This is in line with previous studies showing that SVD-related lesion burden, including white matter lesions, is strongly associated with cognitive and functional impairment [48]. Further, previous studies regarding the impact of white matter changes also reported weak negative correlations between MMSE and Fazekas score especially in patients with AD [49] Although hypothesizing that imaging biomarkers for AD are less informative in very old patients [49] However, no association between brain imaging markers and delirium symptom severity emerged. This is in line with a study of Cavallari and colleagues (2015), who also failed to show an association between delirium incidence and severity and white matter hyperintensities, general and hippocampal atrophy in postoperative older adults without prediagnosed dementia [50].

Similar to previous studies, we could show overall elevated CRP levels in our patients with delirium [8], which supports our finding of infection as the main diagnosis in our sample.

Although infection as etiology for delirium was very prevalent in our examined patients, this should not lead to an underestimation of the general heterogeneity of the origins of delirium.

However, the data fails to show an association between elevated CRP levels and delirium severity and poorer performance in MMSE, which was indicated by previous studies [51] but also contradicted by others [52].

Furthermore, there is evidence for an increased risk of a need of residential care after an episode of delirium due to functional decline especially for patients with dementia [53]. Even though the data of the current study shows a general recovery in patients’ abilities in the basic ADL, the BI at discharge still indicates severe dependency, which is in line with van Roessel and colleagues (2019). We also found a proportionate increase of the need for residential care, although not compared to a control group without delirium and independent of preclinical cognitive status.

### Limitations and strengths

When interpreting the results of the present study the following limitations should be taken into account: 1) The present study is based on observational chart reviews and data were only extracted for patients with delirium without collecting data for a control group, as no group comparison was intended, but rather an evaluation of temporal changes in delirium symptoms similar to Leonard et al. (2013)⁠. To our knowledge, the study of Leonard et al. (2013) is the only one addressing the clinical course of delirium measuring individual symptoms, although in a palliative care setting, where only small changes in individual symptoms over time were detected. 2) No follow-ups (eg. 3, 6 or 12 months) were conducted after patients were released from the hospital, thus results of the present study only suggest/indicate long-term outcomes. 3) The sample size is rather small, but sufficient for statistical analyses. 4) Heterogeneity regarding incidence of delirium, as only the time of initial diagnoses was documented. However, the aim of the present study was the assessment of the clinical course of delirium symptoms and its severity in the non-postoperative setting and only patients with delirium above the predefined cut-off score were included. 5) The lack of significant associations between brain imaging and delirium parameters in our study may also be due to heterogeneity in imaging techniques, as 18 patients received routine CT scans, which are likely to be less accurate and less sensitive for more subtle parenchymal alterations than dedicated MRI.

Strengths of this study include the longitudinal approach assessing the course of delirium in a non-postoperative setting. To our knowledge, this study is one of the only ones conducting a detailed assessment of the course of delirium on a symptom level. In addition, a detailed overview of concurrent clinical characteristics (brain, blood, diagnoses) was collected.

## Conclusion

The mean delirium severity and cognitive functioning at t1 indicate a group-specific vulnerability for patients with delirium superimposed on dementia. However, delirium severity was decreasing over the course of medical treatment independently of symptom, age, sex or preclinical cognitive status. Within one week of treatment the delirium severity fell under the cut-off for diagnosis, even though impairments in short- and long-term memory remained. Severe sleep-wake cycle disturbances are detrimental for cognitive functioning and should be managed using pharmacological and non-pharmacological approaches. Even though the functional abilities of our patients improved over the course of treatment, they remained severely dependent on help at discharge. No associations between brain imaging markers and delirium severity were found, but an impact on cognitive functioning, suggesting limited prognostic value of structural CT and MRI for delirium severity. In addition, no association between CRP/WBC levels and delirium severity and cognitive functioning emerged, also suggesting limited prognostic value.

Given the high incidence and prevalence of delirium in hospitalized older patients and its detrimental impact on cognition, abilities of ADL and personal independence further research regarding risk factors and pathophysiology of delirium needs to be done in order to improve diagnoses and establish prevention programs.

## Supporting information

S1 File Methods supplementary. (DOCX)

S1 Fig Correlation structure of assessment at t1. (TIF)

S2 Fig Correlation structure of assessment at t2. (TIF)

S3 Fig Correlation structure of assessment at t3. (TIF)

S4 Fig Diagnostic plots linear mixed model with DRS-R-98 score. (TIF)

S5 Fig Diagnostic plots linear mixed model with DRS symptoms. (TIF)

S6 Fig Diagnostic plots linear mixed model with MMSE. (TIF)

S1 Table Results of final LME model on DRS-R-98 score. (DOCX)

S2 Table Results of final LME model on DRS-R-98 symptom scores. (DOCX)

S3 Table Results of final LME model on MMSE scores. (DOCX)

S4 Table R-squared values for the final models. (DOCX)
