# Supplementary material for: Delirium in older hospitalized patients—A prospective analysis of the detailed course of delirium in geriatric inpatients
Source: PLoS One. 2023 Mar 16;18(3):e0279763. doi: 10.1371/journal.pone.0279763 (PMC10019648; doi:10.1371/journal.pone.0279763)
Supplement: S1 File — (DOCX) [file pone.0279763.s001.docx]

**Methods Supplementary**

**Brain Imaging Biomarkers**

a) Global cortical atrophy (GCA)

The GCA scale was used to assess cerebral atrophy (Pasquier et al., 1996). ⁠ Thirteen brain regions were assessed separately in each hemisphere. “0” indicating no cortical atrophy and “3” indicating severe atrophy.

b) Medial temporal lobe atrophy (MTLA): Scheltens’ scale

The Scheltens’ scale was used to determine the extent of medial temporal lobe atrophy indicative for Alzheimer's disease (Scheltens et al., 1992).⁠ The following scoring system was applied: “0” meaning no atrophy and “4” meaning severe volume loss of hippocampus.

c) White matter changes: Wahlund scale

White matter and basal ganglia lesions were assessed via Wahlund scale using the following scoring system: “0” indicating no lesions and “3” indicating diffuse involvement of the entire region/confluent lesions (Wahlund et al. 2001).

d) Periventricular hyperintensity (PVH): Fazeka’s scale

White matter lesions (WML) and PVH indicative for vascular dementia and AD were assessed using the Fazeka’s scale (Fazekas et al., 1987).⁠

e) Posterior atrophy: Koedam scale

The Koedam Scale was used to evaluate the extent of posterior atrophy (Koedam et al., 2011).⁠ The posterior cingulate-, parieto-occipital sulcus, the sulci of the parietal lobes and precuneus were rated in the sagittal, axial and coronal orientation using the following scoring system: “0” indicating no significant widening and “3” end-stage atrophy.

**Statistical Methods**

For the linear mixed-effects (LME) models, variables were standardized by Gelman’s recommendation: Binary predictors were not standardized, while numeric predictors were centered and scaled by dividing by two standard deviations (Gelman et al., 2008).⁠ This leads to comparable effect estimates in our linear mixed-effects model regressions.

Following closely the guidelines of Harrison and colleagues for model selection, we started in all three scenarios with a full global model to examine assumptions of a linear mixed-effects model (Harrison et al., 2018).⁠ Afterwards, in the second scenario, the amount of observations allowed us to specify random effects structure using ANOVA, while in the first and third scenario only the random intercept for individual variation was permittable as a random effect. Model selection for fixed effects was done by assessing subsets of combinations of fixed effects using the groups described above. All models with an AIC of the model with the lowest AIC plus 6 were allowed as the top model set. The nesting rule identified the most parsimonious model(s) within the top model set.

First, we investigated the general dynamics of the total DRS-R-98 score. Here, we used the DRS-R-98 score per patient’s assessment as outcome, and explained these scores with an LME using as independent variables the individual subject variation (random effect), time (fixed effect), confounding demographic factors (fixed effects, age, gender, dementia), medication (fixed effects, Melperone, Risperidone, or any of these two), and laboratory parameters (fixed effects, CRP, WBC), as well as interaction between time and all other variables. Supplementary S-Table 2 shows the final model and the effect estimates.

Second, we analyzed the temporal dynamics of individual DRS-R-98 symptoms. To this end, our outcome is the score in points for the respective symptom (between 0 and 3). We employed an LME model with subject variation (random effect), subject-specific random slopes of laboratory parameters CRP and WBC (random effects), symptom-specific effects and symptom-specific temporal effects (fixed effects), confounding demographic factors (fixed effects, age, gender, dementia), medication (fixed effects, Melperone, Risperidone, or any of these two), and laboratory parameters (fixed effects, CRP, WBC), as well as interaction with time for all fixed effects. Supplementary S-Table 3 shows the final model and the effect estimates.

Third, we explored the relationship between MMSE and delirium symptoms as measured by DRS-R-98 rating scale in order to investigate the impact of delirium symptoms on cognition. For this purpose, we used an LME model with MMSE scores at study visits as outcome, with independent variables individual subject variation (random effect), time (fixed effect), delirium symptom scores (fixed effects), confounding demographic factors (fixed effect, age, gender, and dementia), medication (fixed effects, Melperone, Risperidone, or any of these two), and laboratory parameters (fixed effects, CRP, WBC), as well as interaction with time for all fixed effects. Supplementary S-Table 4 shows the final model and the effect estimates.

**Supplementary References**

1. Pasquier F, Leys D, Weerts JGE, Mounier-Vehier F, Barkhof F, Scheltens P. Inter- and intraobserver reproducibility of cerebral atrophy assessment on MRI scans with hemispheric infarcts. *Eur Neurol*. 1996;36(5):268-272. doi:10.1159/000117270.

2. Scheltens P, Leys D, Barkhof F, et al. Atrophy of medial temporal lobes on MRI in “probable” Alzheimer’s disease and normal ageing: diagnostic value and neuropsychological correlates. *J Neurol Neurosurg Psychiatry*. 1992;55(10):967-972. doi:10.1136/jnnp.55.10.967.

3. Fazekas F, Chawluk JB, Alavi A. MR signal abnormalities at 1.5 T in Alzheimer’s dementia and normal aging. *Am J Neuroradiol*. 1987;8(3):421-426. doi:10.2214/ajr.149.2.351.

4. Koedam ELGE, Lehmann M, Van Der Flier WM, et al. Visual assessment of posterior atrophy development of a MRI rating scale. *Eur Radiol*. 2011;21(12):2618-2625. doi:10.1007/s00330-011-2205-4.

5. Gelman A. Scaling regression inputs by dividing by two standard deviations. *Stat Med*. 2008;27(15):2865-2873. doi:10.1002/sim.3107.

6. Harrison XA, Donaldson L, Correa-Cano ME, et al. A brief introduction to mixed effects modelling and multi-model inference in ecology. *PeerJ*. 2018;6:e4794. doi:10.7717/peerj.4794.
