# Supplementary figures and images for: Delirium in older hospitalized patients—A prospective analysis of the detailed course of delirium in geriatric inpatients
Source: PLoS One. 2023 Mar 16;18(3):e0279763. doi: 10.1371/journal.pone.0279763 (PMC10019648; doi:10.1371/journal.pone.0279763)

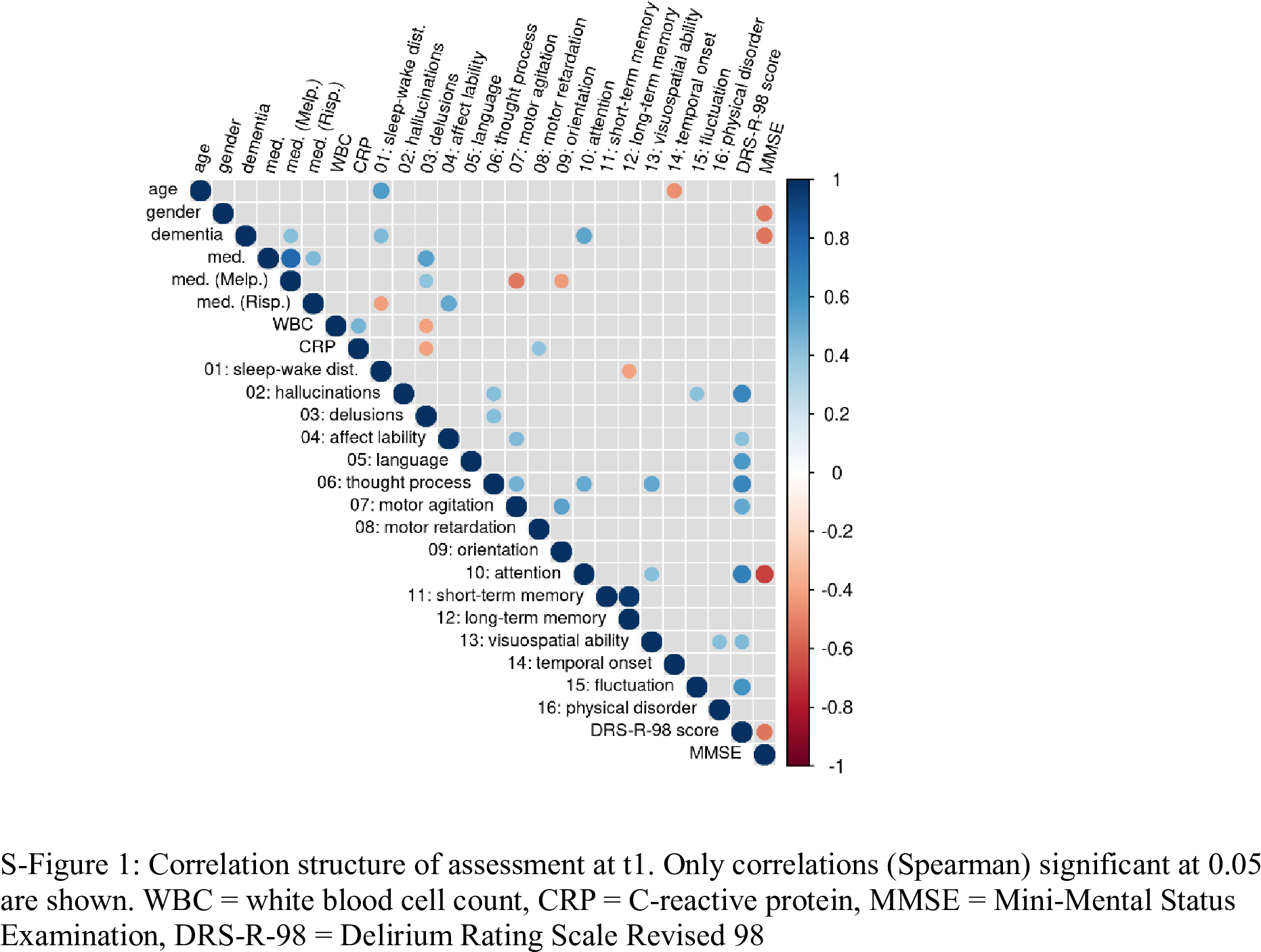

Supplement: S1 Fig — (TIF) [file pone.0279763.s002.tif]

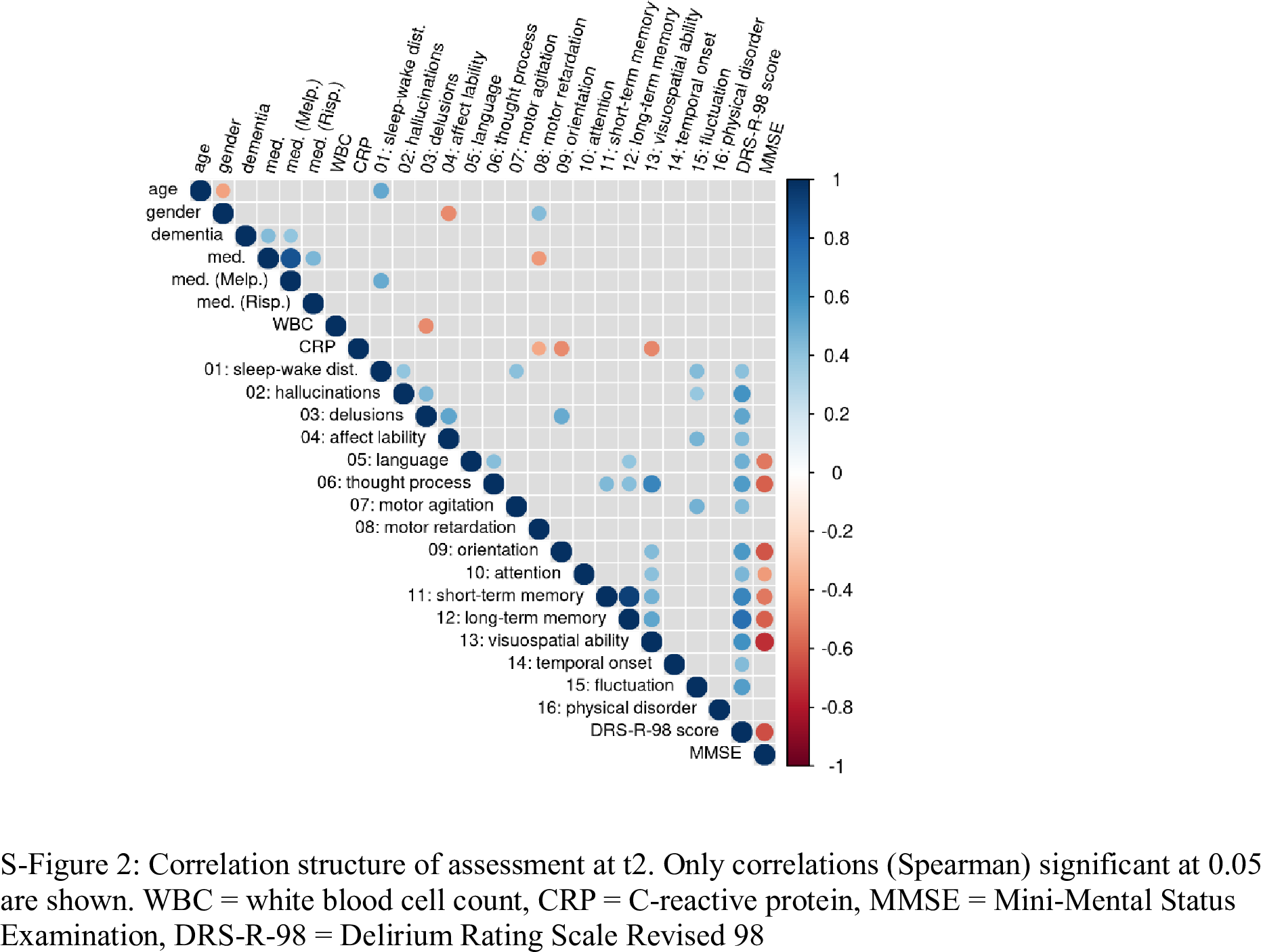

Supplement: S2 Fig — (TIF) [file pone.0279763.s003.tif]

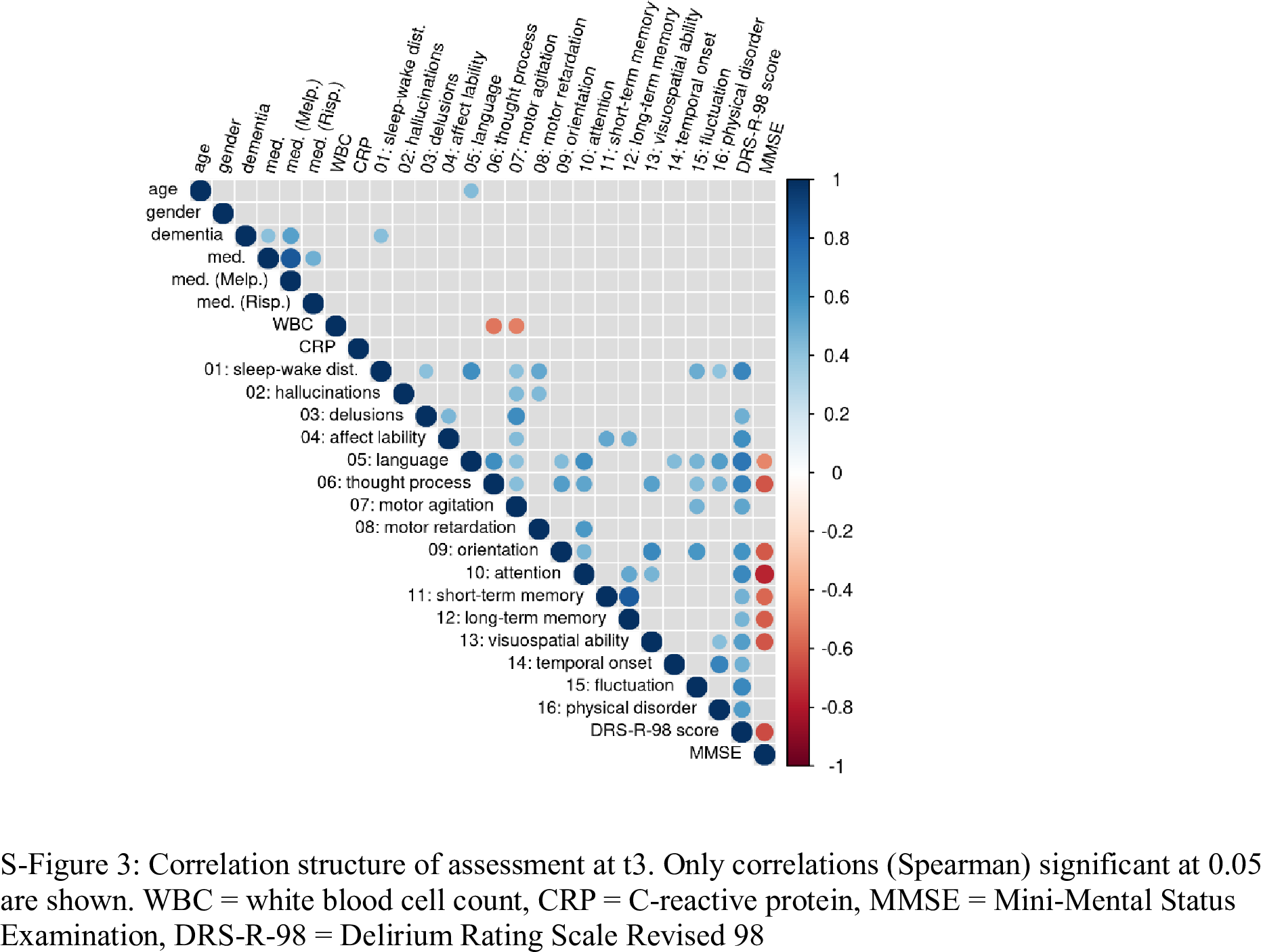

Supplement: S3 Fig — (TIF) [file pone.0279763.s004.tif]

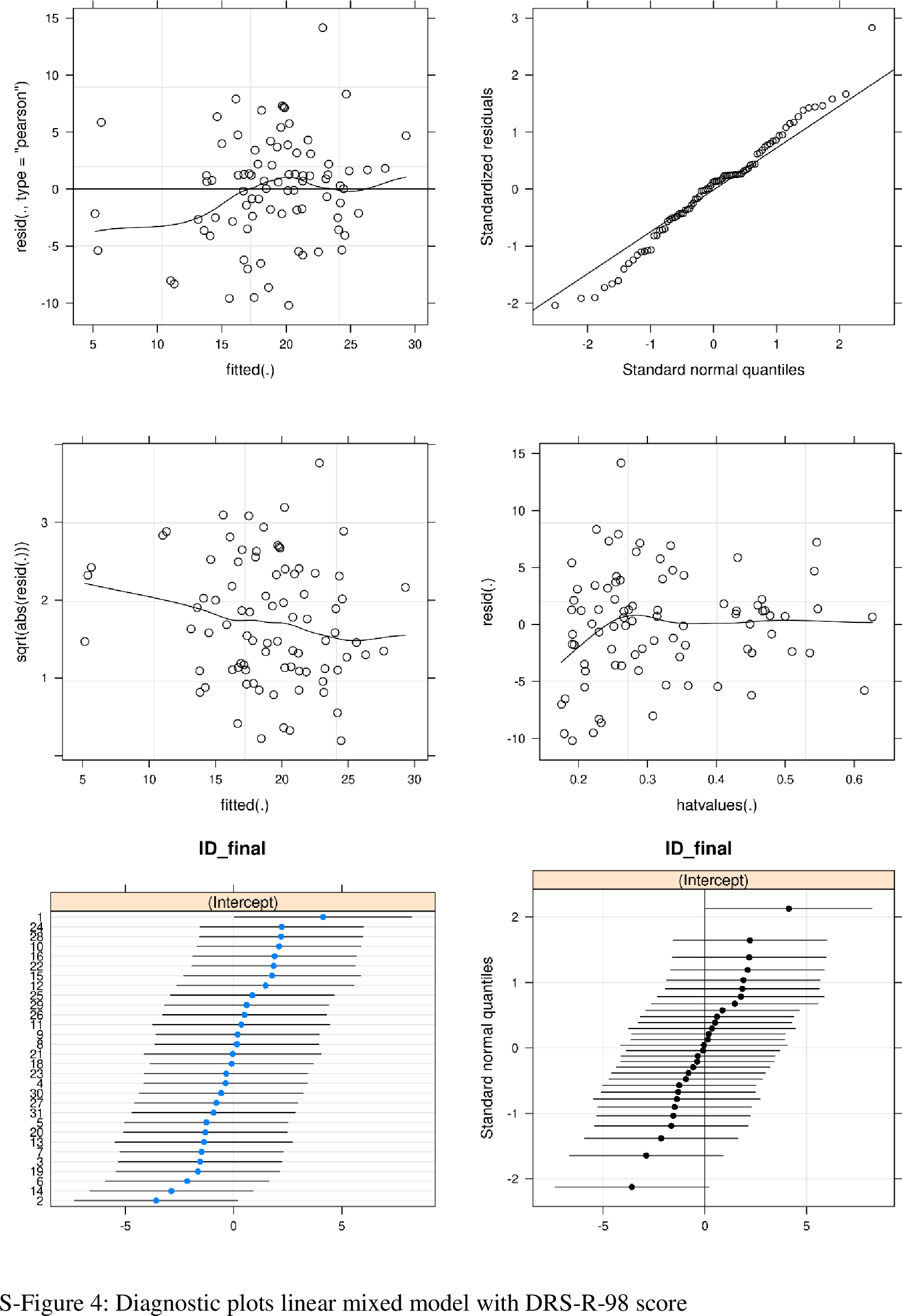

Supplement: S4 Fig — (TIF) [file pone.0279763.s005.tif]

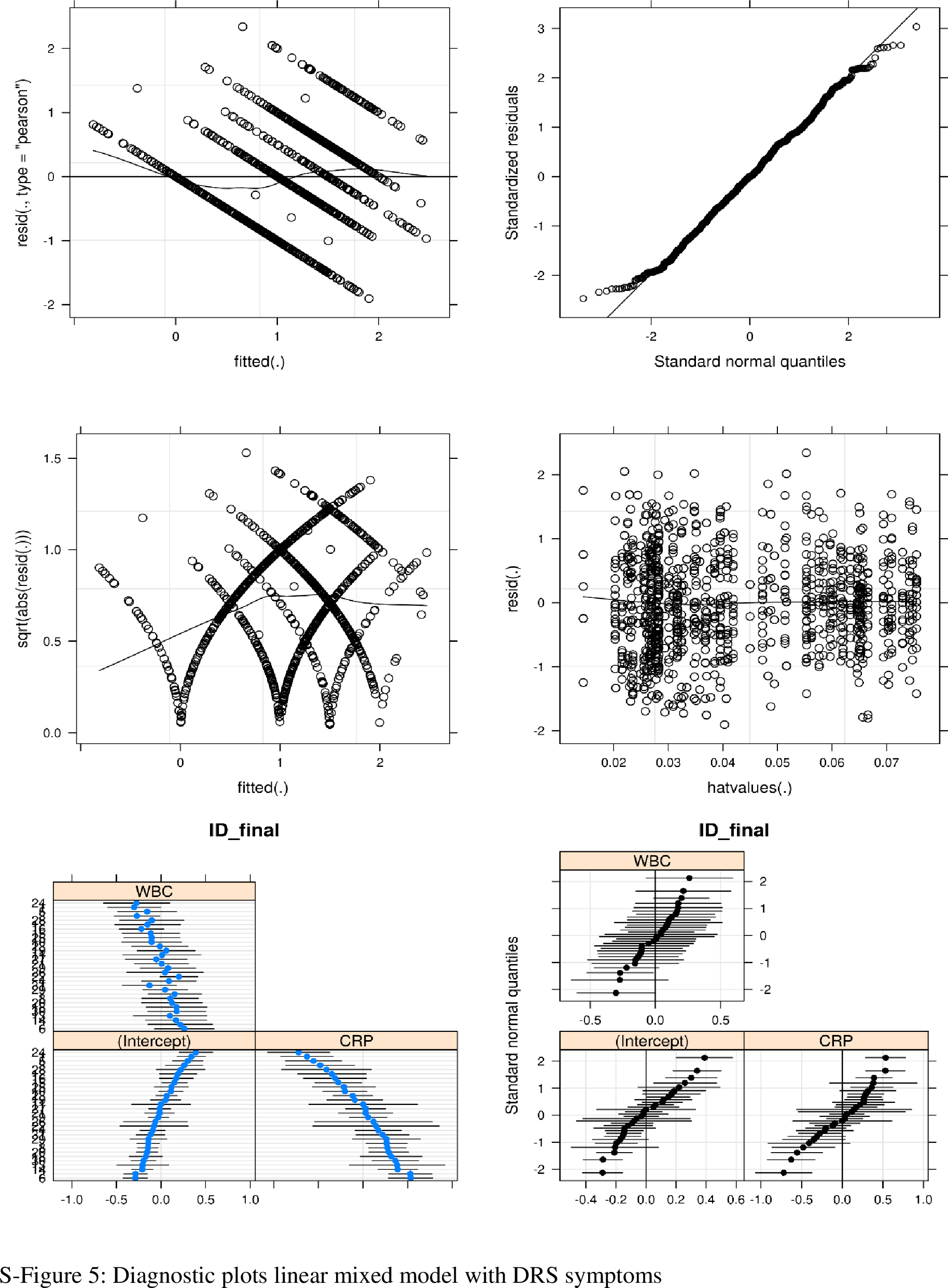

Supplement: S5 Fig — (TIF) [file pone.0279763.s006.tif]

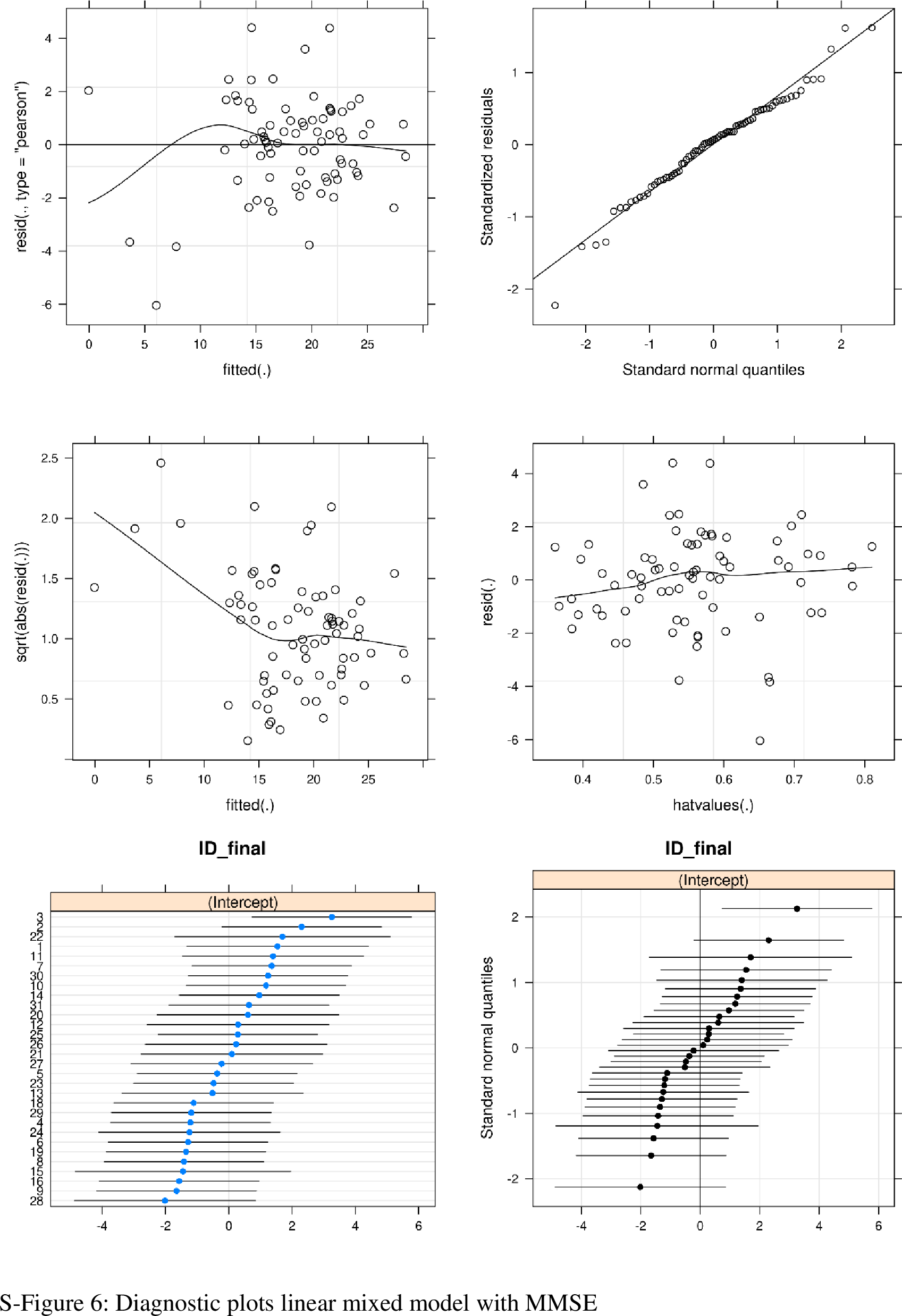

Supplement: S6 Fig — (TIF) [file pone.0279763.s007.tif]
