# Supplementary material for: Delirium in older hospitalized patients—A prospective analysis of the detailed course of delirium in geriatric inpatients
Source: PLoS One. 2023 Mar 16;18(3):e0279763. doi: 10.1371/journal.pone.0279763 (PMC10019648; doi:10.1371/journal.pone.0279763)
Supplement: S1 Table — (DOCX) [file pone.0279763.s008.docx]

S-Table 1: Results of final LME model on DRS-R-98 score

| variable | estimate | CI-95% lower | CI-95% upper | p-value | sign. |
| --- | --- | --- | --- | --- | --- |
| (Intercept) | 23.03 | 20.86 | 25.23 | 0 |  |
| time_day | -1.42 | -1.90 | -0.92 | 0 |  |

Note. LME = Linear Mixed Effects model, DRS-R-98 score = Delirium Rating Scale Revised 98 score, CI = confidence interval, sign. = significance.
