# Supplementary material for: Delirium in older hospitalized patients—A prospective analysis of the detailed course of delirium in geriatric inpatients
Source: PLoS One. 2023 Mar 16;18(3):e0279763. doi: 10.1371/journal.pone.0279763 (PMC10019648; doi:10.1371/journal.pone.0279763)
Supplement: S2 Table — (DOCX) [file pone.0279763.s009.docx]

S-Table 2: Results of final LME model on DRS-R-98 symptom scores

| variable | estimate | CI-95% lower | CI-95% upper | p-value | sign. |
| --- | --- | --- | --- | --- | --- |
| (Intercept) | 1.61 | 1.32 | 1.88 | 0.000 |  |
| attention | 0.23 | -0.11 | 0.56 | 0.187 |  |
| short-term memory | 0.36 | 0.02 | 0.69 | 0.038 |  |
| long-term memory | 0.39 | 0.05 | 0.72 | 0.024 |  |
| visuospatial abilities | 0.11 | -0.22 | 0.45 | 0.509 |  |
| symptom onset | 0.35 | 0.01 | 0.68 | 0.042 |  |
| symptom fluctuation | -0.63 | -0.96 | -0.29 | 0.000 |  |
| physical disease | 0.29 | -0.05 | 0.63 | 0.090 |  |
| hallucination | -0.48 | -0.82 | -0.15 | 0.005 |  |
| delusion | -0.61 | -0.95 | -0.28 | 0.000 |  |
| affect lability | -0.07 | -0.41 | 0.26 | 0.665 |  |
| language | -0.31 | -0.64 | 0.03 | 0.074 |  |
| thought process | 0.21 | -0.12 | 0.55 | 0.211 |  |
| agitation | -0.63 | -0.96 | -0.29 | 0.000 |  |
| motor retardation | -1.13 | -1.47 | -0.79 | 0.000 |  |
| orientation | 0.05 | -0.29 | 0.38 | 0.792 |  |
| sleep-wake cycle:time_day | -0.11 | -0.18 | -0.05 | 0.001 |  |
| attention:time_day | -0.08 | -0.15 | -0.02 | 0.012 |  |
| smemory:time_day | -0.04 | -0.10 | 0.03 | 0.237 |  |
| lmemory:time_day | -0.05 | -0.12 | 0.01 | 0.101 |  |
| visuospatial abilities:time_day | -0.08 | -0.14 | -0.01 | 0.019 |  |
| onset:time_day | -0.06 | -0.13 | 0.01 | 0.073 |  |
| fluctuation:time_day | -0.08 | -0.14 | -0.01 | 0.018 |  |
| disease:time_day | -0.07 | -0.13 | 0.00 | 0.039 |  |
| hallucination:time_day | -0.16 | -0.23 | -0.10 | 0.000 |  |
| delusion:time_day | -0.05 | -0.12 | 0.02 | 0.133 |  |
| affect lability:time_day | -0.11 | -0.18 | -0.05 | 0.001 |  |
| language:time_day | -0.06 | -0.13 | 0.00 | 0.054 |  |
| thought process:time_day | -0.12 | -0.19 | -0.06 | 0.000 |  |
| agitation:time_day | -0.10 | -0.17 | -0.04 | 0.002 |  |
| motor retardation:time_day | -0.05 | -0.11 | 0.02 | 0.168 |  |
| orientation:time_day | -0.14 | -0.21 | -0.08 | 0.000 |  |

Note. LME = Linear Mixed Effects model, DRS-R-98 = Delirium Rating Scale Revised 98, CI = confidence interval, sign. = significance.
