# Supplementary material for: Delirium in older hospitalized patients—A prospective analysis of the detailed course of delirium in geriatric inpatients
Source: PLoS One. 2023 Mar 16;18(3):e0279763. doi: 10.1371/journal.pone.0279763 (PMC10019648; doi:10.1371/journal.pone.0279763)
Supplement: S3 Table — (DOCX) [file pone.0279763.s010.docx]

S-Table 3: Results of final LME model on MMSE scores

| variable | estimate | CI-95% lower | CI-95% upper | p-value | sign. |
| --- | --- | --- | --- | --- | --- |
| (Intercept) | 21.73 | 19.68 | 23.79 | 0.000 |  |
| time_day | -0.69 | -1.20 | -0.22 | 0.017 |  |
| 01: sleep-wake cycle | -1.07 | -3.10 | 0.93 | 0.378 |  |
| 02: hallucinations | 0.06 | -1.57 | 1.86 | 0.953 |  |
| 03: delusions | -0.53 | -2.40 | 1.32 | 0.623 |  |
| 04: affect lability | -0.74 | -2.85 | 1.15 | 0.495 |  |
| 05: language | -2.24 | -4.39 | 0.16 | 0.072 |  |
| 06: thought process | -1.31 | -3.55 | 0.85 | 0.311 |  |
| 07: agitation | 0.95 | -0.88 | 2.64 | 0.355 |  |
| 08: motor retardation | -2.18 | -3.79 | -0.50 | 0.026 |  |
| 09: orientation | -2.72 | -4.63 | -0.71 | 0.017 | ● |
| 10: attention | -0.59 | -2.32 | 1.16 | 0.577 |  |
| 11: short-term memory | 2.55 | -1.07 | 5.94 | 0.215 |  |
| 12: long-term memory | -3.48 | -6.94 | 0.05 | 0.100 |  |
| 13: visuospatial ability | -2.39 | -4.29 | -0.41 | 0.039 | ● |
| 14: symptom onset | 0.82 | -1.09 | 2.71 | 0.445 |  |
| 15: symptom fluctuation | 0.58 | -1.21 | 2.45 | 0.584 |  |
| 16: physical disease | 1.44 | -0.27 | 3.16 | 0.168 |  |
| age | 2.15 | -0.16 | 4.48 | 0.239 |  |
| sex1 | -4.45 | -7.88 | -3.10 | 0.000 |  |
| group_dem1 | -4.65 | -7.14 | -1.06 | 0.018 |  |
| time_day:age | -0.22 | -0.81 | 0.38 | 0.536 |  |
| time_day:sex1 | 0.93 | 0.38 | 1.54 | 0.006 |  |
| time_day:group_dem1 | 0.81 | 0.25 | 1.36 | 0.015 | ● |

Note. LME = Linear Mixed Effects model, MMSE = Mini-Mental Status Examination, DRS-R-98 = Delirium Rating Scale Revised 98, CI = confidence interval, sign. = significance.
