# Supplementary material for: Delirium in older hospitalized patients—A prospective analysis of the detailed course of delirium in geriatric inpatients
Source: PLoS One. 2023 Mar 16;18(3):e0279763. doi: 10.1371/journal.pone.0279763 (PMC10019648; doi:10.1371/journal.pone.0279763)
Supplement: S4 Table — (DOCX) [file pone.0279763.s011.docx]

S-Table 4: R-squared values for the final models

| model | R2 cond. | R2 marg. |
| --- | --- | --- |
| 1: DRS-R-98 total score | 0.225 | 0.452 |
| 2: DRS-R-98 symptoms | 0.291 | 0.421 |
| 3: MMSE | 0.685 | 0.789 |

# *Note.* DRS-R-98 score = Delirium Rating Scale Revised 98 score, MMSE = Mini-Mental Status Examination, R-squared = coefficient of determination, R2 cond. = R-squared conditional, R2 marg. = R-squared marginal.
